# Comparison of root coverage by injectable-platelet rich fibrin in thick and thin phenotypes: a parallel-armed, prospective, preliminary clinical study with up to 48 months of follow-up

**DOI:** 10.1590/1678-7765-2025-0702

**Published:** 2026-04-17

**Authors:** Berceste Guler Ayyildiz, Busra Terzioglu, Seyma Eken

**Affiliations:** 1 Kütahya Health Sciences University Faculty of Dentistry Department of Periodontology Kütahya Turkey Kütahya Health Sciences University, Faculty of Dentistry, Department of Periodontology, Kütahya, Turkey; 2 Kütahya Health Sciences University Tavsanli Vocational School of Health Services Kütahya Turkey Kütahya Health Sciences University, Tavsanli Vocational School of Health Services, Kütahya, Turkey; 3 Kütahya Health Sciences University Oral Health Application and Research Center Kütahya Turkey Kütahya Health Sciences University, Oral Health Application and Research Center, Kütahya, Turkey

**Keywords:** Injectable platelet-rich fibrin, Gingival thickness, Gingival phenotype, Gingival recession, Root coverage

## Abstract

**Objective:**

This study aimed to compare the effectiveness of injectable platelet-rich fibrin (I-PRF) using a submucosal injection technique for creeping attachment in thin and thick phenotypes with gingival recession.

**Methodology:**

The study was designed as a prospective clinical trial. Participants were divided into two groups based on gingival thickness (GT): thin phenotype group (≤1.0 mm) and thick phenotype group (>1.0 mm). I-PRF was applied once a month to each gingival site using the submucosal injection technique, for a total of three applications. Periodontal parameters were measured at baseline (T0), nine months after treatment (T1), and 12–48 months after treatment (T2). Root coverage percentage (RC%) was calculated.

**Results:**

Twelve systemically healthy, non-smoking patients (seven males and five females; mean age: 36.5±9.4), comprising 66 teeth, were analyzed. The primary outcome, RC%, demonstrated a statistically significant intergroup difference at T1 (p=0.032); however, this difference was not statistically significant at T2. In the thick phenotype group, RC% was 20.91±30.80% in T1 and increased to 31.40±30.92% in T2 (p=0.040). Conversely, the thin phenotype group showed higher RC% values at T1 (35.04±29.76%), which were not sustained at T2 (30.70±28.46%; p=0.355). Recession depth decreased in both groups at all time points, but this difference was not statistically significant. Keratinized tissue width showed a significant increase in both groups, from 2.34±1.08 and 2.73±0.95 at T0, respectively, to 2.89±1.17 and 3.28±1.12 at T1 (p<0.05). In the intergroup comparison, GT values were significantly higher in the thick phenotype group than in the thin phenotype group at T0 and T2, while no significant difference was observed at T1.

**Conclusion:**

I-PRF application was shown to enhance RC% in both phenotypes. A significant increase in GT was observed in the thin phenotype; however, these increases in GT and RC% were not fully sustained during long-term follow-up in this sample.Clinical Trial registration: NCT05591326

## Graphical abstract

**Figure:**
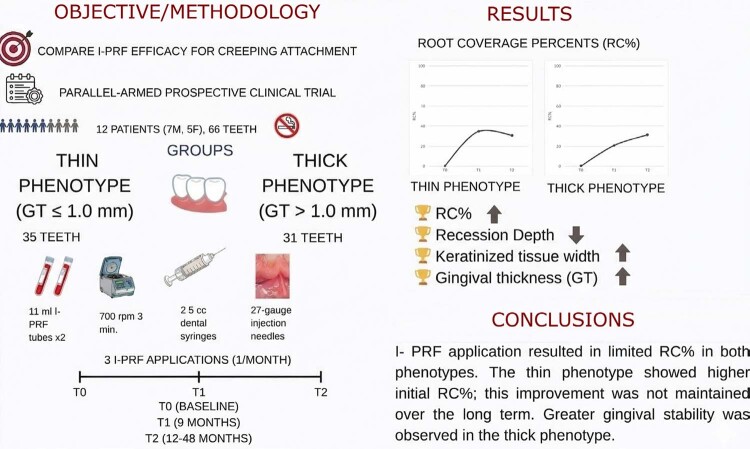

## Introduction

The use of platelet concentrates in medicine and dentistry has become increasingly widespread in recent years, primarily due to their capacity to promote enhanced healing processes.^1^ Platelet-rich fibrin (PRF), a second-generation platelet concentrate, was developed to eliminate the need for anticoagulants required to obtain platelet-rich plasma (PRP), a first-generation platelet concentrate.^2^ It has been demonstrated that PRF demonstrates greater biochemical stability than PRP due to the absence of supplementary anticoagulants. Furthermore, it has been shown to promote faster and more effective wound healing.^3^ In recent years, a new liquid platelet concentrate, injectable platelet-rich fibrin (I-PRF), has been developed using the low-speed centrifugation concept (LSCC).^4^ I-PRF, which contains liquid fibrinogen and thrombin, has gained increasing recognition in dentistry. Its efficacy is attributed to three main factors: ease of application, cost-effectiveness, and a high concentration of growth factors, with a release period of 10–14 days.^5^

The term “gingival phenotype” is a concept derived from the assessment of gingival thickness (GT) and keratinized tissue width (KTW), as defined in the 2017 World Workshop on the Classification of Periodontal and Peri-Implant Diseases and Conditions.^6^ Although the guidelines state that attached gingiva is sufficient to maintain periodontal health when optimal oral hygiene is achieved, thin phenotypes have been reported to be more prone to gingival recession, particularly as a result of inflammatory, traumatic, or surgical interventions.^6-8^ Previous studies have investigated the impact of I-PRF on gingival fibroblasts, its role in modifying the gingival phenotype, and its application in treating gingival recession.^9,10^ Clinical trials have demonstrated that the adjunctive use of I-PRF in periodontal plastic surgery can improve clinical outcomes, particularly in terms of root coverage (RC%), KTW, and recession depth (RD). In a study combining I-PRF with a coronally advanced flap (CAF) and a connective tissue graft (CTG), the addition of I-PRF resulted in greater increases in KTW and reductions in RD compared with CAF and CTG alone.^11^ Moreover, the application of I-PRF as a root surface bio-modifying agent in free gingival graft (FGG) surgery has shown favorable effects on RC%.^12^

Despite the clinical success of mucogingival surgical procedures, the need for minimally invasive treatment alternatives has been emphasized, as postoperative discomfort associated with pain, bleeding, and donor-site morbidity is commonly reported in autogenous soft tissue grafting procedures.^13-15^ A clinical study reported a 29% increase in GT six months after I-PRF application in areas with a thin gingival phenotype. The use of I-PRF alone may offer a significant advantage as a non-surgical treatment option by reducing morbidity compared to conventional soft tissue grafting procedures.^16^ Furthermore, gingival recession coverage has also been achieved with the application of I-PRF in thin-phenotype teeth using a minimally invasive, semi-surgical approach.^10^ However, studies evaluating creeping attachment and RC% following I-PRF application are limited. To our knowledge, RC% associated with I-PRF has not been previously evaluated across different gingival phenotypes.

This study aimed to compare creeping attachment and RC% following I-PRF application in thin and thick phenotypes. The null hypothesis was that the effects of I-PRF on creeping attachment and RC% do not differ between thick and thin phenotypes.

## Methodology

### Trial Design and Setting

This study was a prospective, parallel-arm clinical trial with a follow-up period of 12–48 months. Institutional Review Board (IRB) approval was obtained from the Kutahya Health Sciences University Clinical Research Ethics Committee (Decision No. 2020-04/02). The study was registered on ClinicalTrials.gov under the identifier NCT05591326 on October 19, 2022. The research was conducted in accordance with the Declaration of Helsinki (2013) and Good Clinical Practice guidelines. Personal data of all participants was safeguarded, and every participant signed an informed consent form. The study was designed in accordance with the CONSORT 2025 guidelines.^17^ It was conducted at the Faculty of Dentistry, Periodontology Department, at Kutahya Health Sciences University, between 2021 and 2025.

### Eligibility Criteria

Patient-related inclusion criteria were as follows: (1) patients aged between 18 and 65 years; (2) absence of systemic disease; and (3) ability to cooperate during clinical procedures.

Tooth/site-related inclusion criteria were as follows: (1) absence of interproximal tissue loss, classified as Cairo Recession type 1 (RT1);^18^ (2) teeth without root surface caries and/or restorations; (3) absence of non-carious cervical lesions and/or recession defects associated with pulp pathology; and (4) no history of mucogingival surgery in the relevant area.

Exclusion criteria were as follows: (1) pregnancy and breastfeeding; (2) individuals with intellectual disabilities; (3) heavy smoking (>10 cigarettes/day); (4) patients with bleeding disorders or those using anticoagulant medications; (5) patients taking medications that suppress the immune system or impair healing; (6) mucogingival stress or bruxism; (7) active orthodontic treatment; and (8) untreated periodontitis (full-mouth plaque score > 20%, full-mouth bleeding score >25%, residual pocket depth >5≥mm).

Patients were divided into two groups according to gingival phenotype:

- Thin phenotype: Buccal gingival thickness ≤ 1.0 mm^19^
- Thick phenotype: Buccal gingival thickness > 1.0 mm^19^

### Intervention and Comparator

The same periodontist (B.G.A.) performed the I-PRF procedure on all patients using a standardized protocol. Venous blood samples were collected from each patient into two 11-ml I-PRF tubes (BD Vacutainer^®^, NJ, USA) without anticoagulant using the Vacusera^®^ blood collection system (Disera, Izmir, Turkey), which included a 21-gauge butterfly needle and adapter. The collected blood samples were centrifuged at 700 rpm (60 × g) for three minutes at room temperature using a centrifuge device (Nuve, NF 800 R, Ankara, Turkey).^20^ The resulting I-PRF was transferred to 2.5 cc dental syringes (Berika, Konya, Turkey) using a 21-gauge needle. Local anesthesia (Ultracaine DS Forte, Sanofi-Aventis, Germany) was administered to the treatment area. The prepared I-PRF was injected using 27-gauge dental injection needles into the apical mucogingival junction of the keratinized gingival area. I-PRF application was performed three times, at one-month intervals.

Prior to the initiation of the study, a calibration process was conducted for the clinical measurements performed by two researchers (B.T. and S.E.) to ensure measurement reliability and precision. Linear parameters (e.g., RD, KTW, and GT) were measured twice, with a two-week interval, in 10 patients who were not included in the study. Intraclass correlation coefficient (ICC) values for intra-examiner reliability ranged between 0.984 and 0.993 for the evaluated clinical parameters. Furthermore, ICC values for inter-examiner reliability were >0.96 for all linear measurements.

The clinical measurements were conducted by two calibrated researchers (B.T. and S.E.) using a standard William-type periodontal probe (Nordent Manufacturing Inc., Elk Grove Village, Illinois, USA). Measurements were recorded before the I-PRF procedure (T0), nine months after the procedure (T1), and 12–48 months after the procedure (T2). The assessed parameters included probing depth (PD), plaque index (PI),^21^ gingival index (GI),^22^ and the percentage of bleeding on probing (BOP%) at six sites per tooth: mesio-buccal, mid-buccal, disto-buccal, mesio-palatal/lingual, mid-palatal/lingual, and disto-palatal/lingual, at all time points. Furthermore, the following measurements were recorded at all time points: KTW, GT, RD, recession width (RW), and vestibular depths (VD). KTW in the buccal region of the tooth was measured in millimeters from the mucogingival junction to the gingival margin. GT was measured using a transgingival probing technique. After the administration of local anesthesia, a #15 endodontic finger spreader equipped with a rubber stopper was inserted perpendicularly through the mid-buccal gingiva, approximately 1.5 mm apical to the gingival margin of each tooth, until root resistance was encountered. The rubber stopper was then gently brought into contact with the gingiva to avoid tissue compression. VD was measured from the gingival margin to the deepest concavity of the mucobuccal fold. The distance between the instrument tip and the stopper was subsequently measured in millimeters using a digital caliper. RC% was calculated using the following formula “

( Preoperative RD -Postoperative RD)/Preoperative RD×100”

(Figure 1).

**Figure 1:**
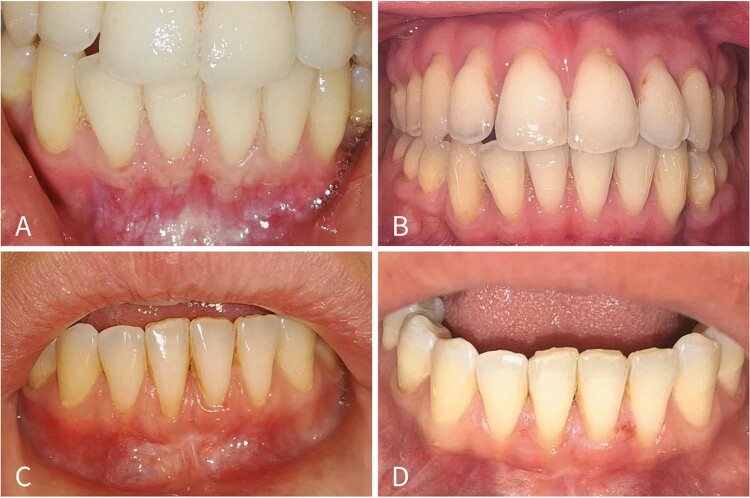
Clinical photographs before I-PRF application and at follow-up.A. Thin phenotype group at T0. B. Thin phenotype group at T2. C. Thick phenotype group at T0 D. Thick phenotype group at T2.

### Outcomes

Primary outcomes: Comparison of creeping attachment and RC% after I-PRF application in inter- and intra-group analyses.

Secondary outcomes: (1) Comparison of KTW, GT, and clinical periodontal parameters after I-PRF application in inter- and intra-group analyses. (2) Evaluation of the effects of demographic and clinical parameters on RC%.

### Sample Size

The main hypotheses of the study were designed to investigate differences between independent groups and changes over time. Similar studies suitable for sample size calculation were reviewed, and the sample size yielding the largest estimate, based on the statistical methods aligned with the main hypotheses, was selected. In this study, the sample size was calculated using the G. Power-3.1.9.2 program (Franz Faul, Germany). A standardized effect size of 0.8210, representing the difference between CAF+A-PRF and CAF, was obtained from a similar study at a 95% confidence level (a=0.05).^23^ A minimum sample size of 25 per group and time point was calculated, with a theoretical power of 0.80. Considering the possibility of loss of observations over time, an additional 40% of the calculated sample size (10 observations) was included. Thus, the minimum required sample size was determined to be 35.

### Blinding

All participants and the statistician were blinded to phenotype groups. However, the researchers who administered the I-PRF and performed the measurements were not blinded, as the study protocol required direct measurement of GT thickness at all time points.

### Statistical Methods

Descriptive statistics (mean, standard deviation, minimum, maximum, and frequency distribution) were calculated for the data. According to the Kolmogorov–Smirnov test results, the data did not follow a normal distribution; therefore, comparisons of clinical data between thick and thin phenotype groups were performed using the Mann–Whitney U test. The Friedman test was used to examine differences across three time points, despite the assumption of normality not being met. Post hoc Bonferroni-corrected tests were performed to determine which time points contributed to the significant differences. Linear regression analysis was conducted using a stepwise approach to model the relationship between a continuous dependent variable and independent variables. Analysis of variance (ANOVA) was applied to test the significance of the model. All statistical analyses were performed using IBM SPSS Statistics 25 (Armonk, NY, USA).

## Results

### Participant Flow and Demographic Data

**Figure 2:**
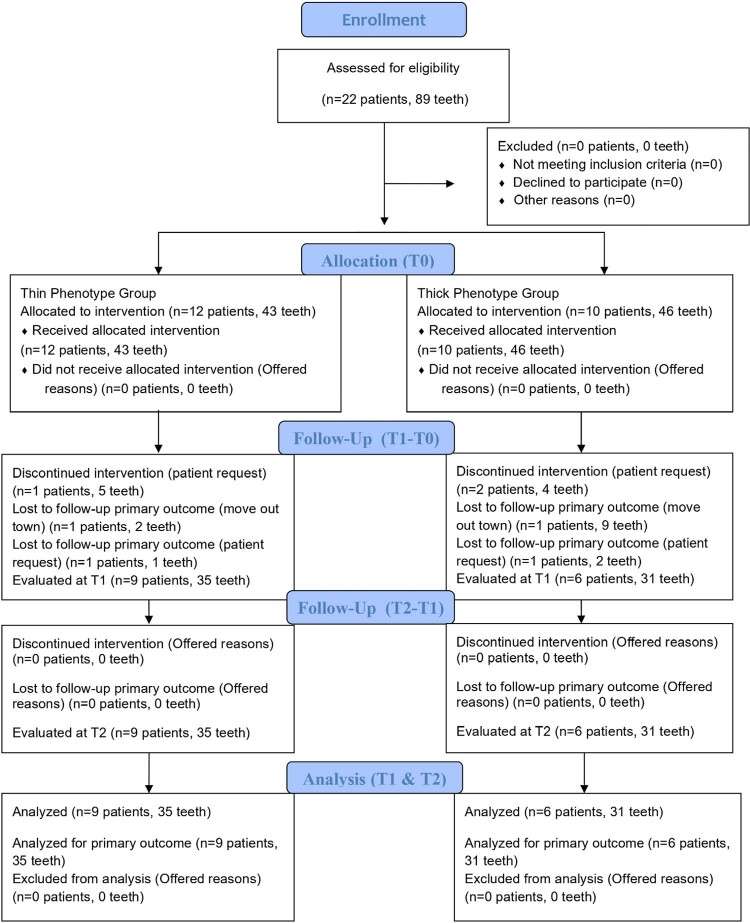
CONSORT 2025 flow diagram.

The study initially included 22 patients and 89 teeth. Two patients elected to withdraw from the treatment regimen, while two others failed to attend their scheduled follow-up appointments. Moreover, two patients could not be contacted due to changes in their addresses. The final analysis encompassed a total of 12 patients (seven males and five females) and 66 teeth. The mean age of the patients was 36.50±9.38 years (min−max: 21–58). No statistically significant age difference was observed between the thin phenotype (37.8±5.93) and thick phenotype (35.0±16.22) groups (p=0.109). All patients were systemically healthy and nonsmokers. The CONSORT study flow diagram is presented in Figure 2.

The distribution of teeth that underwent I-PRF application in the thin and thick phenotype groups is presented in Table 1. In both groups, most teeth were located in the mandible (65.7% and 64.5%, respectively). While 42.9% of the teeth in the thin phenotype group were located in the anterior region, this ratio was 58.1% in the thick phenotype group. The mean follow-up period in the thin phenotype group was 37.23±3.87 months (min−max: 18–41), while it was 39.26±10.83 months (min−max: 12–48) in the thick phenotype group. The mean follow-up period for all teeth was 38.18±7.94 months (min−max: 12–48) (Table 2).

**Table 1:** Comparison of descriptive parameters between gingival phenotype groups

	Thin Phenotype	Thick Phenotype	Total	*p*
	**(n=35)**	**(n=31)**	**(n=66)**	
Maxilla	12 (34.3%)	11 (35.5%)	23 (34.8%)	0.919
Mandible	23 (65.7%)	20 (64.5%)	43 (65.2%)	
Anterior	15 (42.9%)	18 (58.1%)	33 (50.0%)	0.218
Posterior	20 (57.1%)	13 (41.9%)	33 (50.0%)	

*p<0.05 Chi-square test

**Table 2:** Distribution of mean follow-up times between the groups

Groups	n	Min–Max	Follow-up (Months) Mean±SD
**Thin Phenotype**	**35**	**18–41**	**37.23±3.87**
Thick Phenotype	31	12–48	39.26±10.83
Total	66	12–48	38.18±7.94

### Intergroup and Intragroup Comparisons of RC%

Statistically significant differences were observed in both intragroup and intergroup comparisons of RC% (p<0.05). In the intergroup comparison, a statistically significant difference in RC% values between the thin and thick phenotypes was observed at T1 (p=0.032), while no significant difference was found between the two groups at T2 (p=0.720) (Table 3).

**Table 3:** Intra- and inter-group comparison of root coverage percentage over time

Groups	T1 RC% Mean ±S.D.	T2 RC% Mean ±S.D.	p^**^
Thin Phenotype	35.04±29.76	30.70±28.46	0.355
Thick Phenotype	20.91±30.8	31.40±30.92	0.040
*P*	0.032	0.720	

*p<0.05. Mann–Whitney U Test. **Wilcoxon signed-rank test. RC%: Root coverage percentage. T1: Nine months after the procedure. T2: 12–48 months after the procedure.

Statistically significant differences were also observed in intragroup comparisons (p<0.05) (Table 3). In the thin phenotype group, RC% was higher at T1 (35.04±29.76%) than at T2 (30.70±28.46%), but this difference was not statistically significant (p=0.355). In the thick phenotype group, RC% was statistically significantly higher at T2 (31.40±30.92%) than at T1 (20.91±30.8%) (p=0.040).

### Intergroup and Intragroup Comparisons of KTW, GT, RD, RW, and Periodontal Parameters

In both intergroup and intragroup comparisons, statistically significant differences were found between the thin and thick phenotype groups for the KTW, GT, and RD parameters (p<0.05). At T0, KTW was significantly lower in the thin phenotype group (2.34±1.08) than in the thick phenotype group (2.73±0.95); however, no significant differences were observed between the two groups at T1 and T2. GT was statistically significantly higher in the thick phenotype group than in the thin phenotype group at both T0 and T2. No statistically significant differences were found between the thin and thick phenotype groups for RD and RW at T0, T1, or T2 (p>0.05) (Table 4).

**Table 4:** Intra- and inter-group comparison of clinical parameters at different time

Groups	Mean±SD. T0	Mean±SD. T1	Mean±SD. T2	p^**^ (Bonferroni)		
				T0–T1	T0–T2	T1-T2
KTW Thin Phenotype	2.34±1.08	2.89±1.17	2.73±1.18	0.003	0.003	1.000
Thick Phenotype	2.73±0.95	3.28±1.12	3.08±0.89	0.000	0.099	0.633
*p*	0.037	.177	.136			
GT Thin Phenotype	0.67±0.30	0.98±0.47	0.78±0.27	0.009	0.081	0.222
Thick Phenotype	1.11±0.33	1.15±0.43	1.23±0.37	1.000	1.000	1.000
*P*	0.000	.111	.000			
RD Thin Phenotype	1.57±0.78	1.29±0.69	1.17±0.76	0.021	0.024	1.000
Thick Phenotype	1.61±0.76	1.10±0.85	1.06±0.71	0.000	0.003	1.000
*P*	.941	.168	.462			
RW Thin Phenotype	2.03±0.87	1.80±0.77	2.26±1.22	0.057	1.000	0.351
Thick Phenotype	1.79±0.80	1.17±1.03	1.64±1.20	1.000	0.435	1.000
*P*	.188	.596	.081			
PI Thin Phenotype	0.91±0.61	1.10±0.70	0.41±0.52	0.138	0.138	0.090
Thick Phenotype	0.52±0.63	0.48±0.57	0.20±0.35	1.000	0.132	0.084
*P*	0.009	.001	.103			
GI Thin Phenotype	1.11±0.47	0.84±0.52	0.39±0.52	0.024	0.000	0.006
Thick Phenotype	1.00±0.37	0.84±0.37	0.01±0.05	0.075	0.000	0.000
*P*	.923	.923	.000			
PD Thin Phenotype	1.40±0.40	1.43±0.37	1.93±0.70	0.741	0.000	0.000
Thick Phenotype	1.49±0.58	1.55±0.55	1.28±0.33	0.660	1.000	0.408
*P*	.931	.569	.000			
BOP% Thin Phenotype	31.43±47.1	16.13±37.39	5.47±19.81	0.042	0.009	0.222
Thick Phenotype	22.65±42.47	16.13±37.39	0.00±0.00	0.315	0.069	0.471
*p*	.700	1.000	.112			
VD Thin Phenotype	9.19±1.89	9.37±1.88	9.94±2.18	0.033	0.033	0.081
Thick Phenotype	7.92±1.90	8.23±2.05	8.92±3.01	0.015	0.018	0.144
*p*	.017	.043	.067			

*p<0.05. Mann–Whitney U Test. **Wilcoxon signed-rank test. KTW: Keratinized tissue width, GT: Gingival thickness, RD: Recession depth, RW: Recession width, PI: Plaque index, GI: Gingival index, PD: Probing depth, BOP%: Bleeding on probing, VD: Vestibular depths, T0: Before the I-PRF procedure T1: Nine months after the procedure T2: 12–48 months after the procedure.

In intragroup comparisons, KTW was significantly lower in the thin phenotype group at T0 (2.34±1.08) than at T1 (2.89±1.17) and T2 (2.73±1.18). Similarly, KTW was significantly lower in the thick phenotype group at T0 (2.73±0.95) than at T1 (3.28±1.12). In the thin phenotype group, GT was significantly lower at T0 (0.67±0.30) than at T1 (0.98±0.47) (p=0.009). In the thick phenotype group, no statistically significant differences were found in intragroup comparisons (p>0.05). In the thick phenotype group, RD was significantly higher at T0 (1.61±0.76) than at T1 (1.10±0.85) and T2 (1.06±0.71) (p=0.000, p=0.003). No statistically significant differences were found in the intragroup comparison of RD in the thin phenotype group (p>0.05) (Table 4).

Statistically significant differences were observed in both intragroup and intergroup comparisons of periodontal parameters (p<0.05). In intergroup comparisons, the mean PI was significantly higher in the thin phenotype group than in the thick phenotype group at T0 and T1; however, no significant difference was observed between the two groups at T2. The mean GI and PD were significantly lower in the thick phenotype group than in the thin phenotype group only at T2. No significant differences in BOP% were found between the two groups at T0, T1, or T2. The mean VD was significantly higher in the thin phenotype group than in the thick phenotype group at T0 and T1; however, no significant difference was observed between the two groups at T2 (Table 4).

In intragroup comparisons, GI was significantly lower at T2 than at T0 and T1 in both groups (p<0.05). In the thin phenotype group, PD was statistically significantly higher at T2 (1.93±0.70) than at T0 (1.40±0.40) and T1 (1.43±0.37), whereas no significant differences were observed between time points in the thick phenotype group (p>0.05). In the thin phenotype group, BOP% was significantly higher at T0 than at T2 (p=0.009); however, no significant differences were observed between time points in the thick phenotype group (p>0.05). A statistically significant increase in VD was observed at T0 compared to T1 and T2 in both groups (p=0.003, p=0.003, p=0.015, p=0.018) (Table 4).

### Linear Regression analyses for RC%

The linear regression analysis evaluating the effects of other parameters on RC% at T1 is presented in Table 5. The independent variables RD at T1 and KTW at T1 were found to affect RC% (p<0.05). ANOVA was used to assess model significance, and the regression model was found to be significant (F=20.665, p<0.001). The independent variables in the model explained 39.2% of the variance in the dependent variable. Examination of the variance inflation factor (VIF) values for the significant variables in the model showed that all VIFs were below 5, indicating no evidence of multicollinearity or autocorrelation (Durbin–Watson statistic: 1.707 < 2.5).

**Table 5:** Linear regression analysis for RC% at T1

Variables	β	SE	t	*p*
Constant	27.916	10.936	2.553	0.013*
RD	-20.894	4.093	-5.105	<0.001*
KTW	8.064	2.744	2.939	0.005*
Variables not included in the model	β	t	*p*	
GT	0.034	0.329	0.744	
RW	-0.037	-0.280	0.780	
Phenotype	0.194	1.965	0.054	
PI	-0.047	-0.453	0.652	
GI	-0.004	-0.043	0.966	
PD	0.052	0.516	0.608	
BOP%	0.121	1.200	0.235	
Gender (Male)	0.093	0.920	0.361	
Age	-0.040	-0.389	0.699	
Location (Mandible)	0.106	0.848	0.400	
Location (Anterior)	-0.127	-1.242	0.219	
Follow-up period (months)	0.018	0.180	0.858	
F:20.665, p<0.001*	**R^2^:0.412, Adjusted R^2^:0.392**			

*p<0.05, β: Regression coefficient, SE: Standard error. RC%: Root coverage percentage, KTW: Keratinized tissue width, GT: Gingival thickness, RD: Recession depth, RW: Recession width, PI: Plaque index, GI: Gingival index, PD: Probing depth, BOP%: Bleeding on probing, T1: Nine months after the I-PRF procedure.

The regression coefficient for RD was -20.894, and was statistically significant (p<0.05). A one-unit increase in RD at T1 was associated with a decrease of 20.894 units in the mean RC% at T1. The regression coefficient for KTW at T1 was 8.064 and was statistically significant (p<0.05). A one-unit increase in the KTW at T1 was associated with an increase of 8.064 units in the mean RC% at T1 (Table 5).

The linear regression analysis investigating the effects of other parameters on RC% at T2 is presented in Table 6. The independent variables RD at T2 and location (anterior) were found to affect RC% at T2 (p<0.05). ANOVA was used to assess model significance, and the regression model was found to be significant (F=12.025, p<0.001). The independent variables in the model explained 25.3% of the variance in the dependent variable. Examination of VIF values for the significant variables in the model showed that all VIFs were below 5, indicating no evidence of multicollinearity or autocorrelation (Durbin–Watson statistic: 1.965 < 2.5).

**Table 6:** Linear regression analysis for RC% at T2

Variables	β	SE	t	*p*
Constant	59.991	6.723	8.924	<0.001*
RD	-19.598	4.396	-4.458	<0.001*
Location (Anterior)	-14.860	6.274	-2.369	0.021*
Variables not included in the model	β	t	*p*	
KTW	0.110	0.990	0.326	
GT	0.089	0.818	0.416	
RW	-0.061	-0.460	0.647	
Phenotype	0.173	1.617	0.111	
PI	0.133	1.203	0.233	
GI	0.052	0.483	0.631	
PD	-0.125	-1.102	0.275	
BOP%	0.010	0.092	0.927	
Gender (Male)	0.160	1.470	0.147	
Age	-0.010	-0.091	0.928	
Location (Mandible)	0.014	0.131	0.897	
Follow-up period (months)	0.066	0.541	0.590	
F:12.025, p<0.001*	**R^2^:0.276, Adjusted R^2^:0.253**			

*p<0.05, β: Regression coefficient, SE: Standard error. RC%: Root coverage percentage, KTW: Keratinized tissue width, GT: Gingival thickness, RD: Recession depth, RW: Recession width, PI: Plaque index, GI: Gingival index, PD: Probing depth, BOP%: Bleeding on probing, T2: 12–48 months after the I-PRF procedure.

The regression coefficient for RD at T2 was -19.598 and was statistically significant (p<0.05). A one-unit increase in RD at T2 was associated with a decrease of 19.598 units in the mean RC% at T2. The regression coefficient for location (anterior) was -14.860 and was statistically significant (p<0.05). Teeth located in the anterior region showed a 14.860-unit lower mean RC% compared to those located in the posterior region (Table 6).

## Discussion

This study aimed to compare the RC% achieved following I-PRF application in thin and thick gingival phenotypes. The primary outcome indicated that RC% was higher in the thin phenotype group than in the thick phenotype group during the early period; however, similar RC% values were observed between the two groups in the long term. Accordingly, the null hypothesis stating that the effect of I-PRF on creeping attachment and RC% would not differ between thick and thin phenotypes in the early period was partially rejected, whereas in the long term it was partially accepted.

The key advantages of non-surgical I-PRF application include ease of application, the absence of a secondary wound site, and high patient satisfaction. These factors position it as a significant conservative treatment option, particularly for patients with contraindications to surgery. However, it should be noted that I-PRF administration alone cannot be considered a sufficient alternative to the treatment approaches generally regarded as the gold standard for gingival recession. A recent systematic review demonstrated that PRF application may increase the mean RC% when used in combination with CAF.^27^ Alan, et al.^10^ (2023) reported that significant RC% was achieved with semi-surgical I-PRF application in individuals with thin phenotypes. In contrast, Ozsagir, et al.^20^ (2020) reported no change in RC%; however, their study included only teeth with thin phenotypes and had a follow-up period of six months. In this study, the RC% obtained using only I-PRF was limited in both groups. During the long-term follow-up period, a slight, non-significant decrease in RC% was observed in the thin phenotype group, while a significant increase was recorded in the thick phenotype group. Specifically, while RC% showed a minor, non-significant reduction in the thin phenotype group from T1 to T2, a significant increase was observed in the thick phenotype group. The limited decrease in RC% observed in the thin phenotype group, compared to the long-term stability and improvement in the thick phenotype group, highlights the importance of gingival phenotype. This difference may be attributed to the greater volume of connective tissue and higher fibroblast activity inherent in thick phenotypes, which may promote better creeping attachment and long-term stability.^25^ Furthermore, to our knowledge, there is currently a lack of direct comparative studies evaluating standalone I-PRF versus its use in combination with surgical procedures.

Previous studies investigating I-PRF have primarily focused on GT increase and gingival phenotype modification. Significant increases in GT have generally been reported when I-PRF is combined with microneedling (MN).^20,26-28^ Additionally, Manasa, et al.^16^ (2023) reported that after a single session of I-PRF treatment, GT increased by 26.56% at three months and by 29% at six months compared to baseline values. In another study comparing FGG with I-PRF combined with MN applied over three treatment sessions, similar increases in GT were reported in both groups.^29^ Furthermore, a study comparing hyaluronic acid and I-PRF applied to teeth with thin phenotypes reported a significant increase in GT in both groups.^30^ In this study, MN was not used in combination with I-PRF because the primary objective was to evaluate the effect of I-PRF alone. A recent study demonstrated a significant positive correlation between gingival thickness and connective tissue thickness.^31^ The increased interdigitation between epithelial rete pegs and connective tissue papillae, which is typically observed in thicker gingiva, may enhance biomechanical stability. Consequently, this structural characteristic may enable the tissue to exhibit greater resistance and stability in response to regenerative stimuli.^32^ This study revealed an increase in GT in both thin and thick phenotype groups. However, during the long follow-up period, a slight decrease was observed in the thin phenotype group, whereas it remained more stable in the thick phenotype group. These findings may be explained by biological differences between gingival phenotypes. Therefore, increasing the number of I-PRF applications beyond three sessions at one-month intervals may be considered for patients with thin phenotypes in future studies.

Regarding KTW, several studies have reported that I-PRF application has no significant effect on KTW increase.^16,20,33,34^ In contrast, other studies have demonstrated that I-PRF may be effective in increasing KTW.^20,25,29,30^ In this study, a significant increase in KTW was observed in both groups at T1. Furthermore, in the thin phenotype group, KTW remained significantly higher at T2 compared to baseline. However, a slight decrease at T2 compared to T1 was observed in both groups, although this difference was not statistically significant.

A recent systematic review reported that KTW was a significant factor influencing the increase in RC% achieved with CAF combined with PRF.^27^ Consistent with these findings, this study demonstrated a significant effect of KTW on RC% in the regression analysis performed to evaluate factors influencing RC% at T1. Previous research investigating the effect of tooth location on RC% in CAF procedures reported higher mean RC% values in anterior teeth compared to posterior teeth.^35^ Additionally, creeping ridges has been reported to occur more frequently in anterior teeth.^36^ However, in this study, despite a comparable sample size in the anterior and posterior regions, tooth location had no effect on RC% at T1, while anterior localization was found to negatively impact RC% in the long term. This finding may be attributed to the decline in RC% observed in the thin phenotype group. Moreover, in contrast to findings reported in the literature, the absence of surgical intervention in this study may also explain the negative effect of localization on RC%. Factors that may contribute to tissue tension, such as frenulum attachments, were not evaluated, which represents a limitation of this study. The absence of surgical intervention to address such factors may partly explain the observed results.

Recent studies have reported significant reductions in PI and GI values around teeth treated with I-PRF.^20,30,34^ In this study, a significant difference in PI values was observed between the groups in the short term and at baseline. PI values were lower in the thick phenotype group in the short term, but similar results were found in both groups in the long term. A decrease in PI values was also observed in the long term, although this change was not statistically significant. GI values decreased in both groups over time, while PD showed a significant increase only in the thin phenotype group. This outcome can be explained by the patients’ participation in supportive periodontal treatment. Consequently, the favorable outcomes observed for GI and BOP% may not be attributed solely to improved oral hygiene but also to biological characteristics of I-PRF and/or the increase in GT. Despite the comparable selection of study groups and the overall improvement in clinical parameters over time, more favorable clinical outcomes were predominantly observed in the thick phenotype group. The inability to maintain the short-term improvements over the long term in the thin phenotype group may be related to less favorable clinical parameters compared to the thick phenotype group. Additionally, VD increased significantly in both groups, which may be related to the reduction in RD and the coronal shift of the reference point used for measurement. Previous studies have generally applied I-PRF at intervals of seven to ten days, based on the release profile of growth factors.^20,26,27,30^ These studies primarily reported short-term results, whereas this study included a relatively long follow-up period, enabling the evaluation of short- and long-term changes.^20,23^ In this study, three I-PRF applications were performed at one-month intervals. This protocol aimed to provide periodic boosts by administering I-PRF at comparatively longer intervals, thereby enhancing patient compliance and tolerability. Furthermore, while previous studies generally focused on thin phenotypes, this study compared the responses of both thin and thick phenotypes to I-PRF treatment.^10,27^

A notable limitation of this study is that the final sample size did not fully reach the initially calculated target power. Consequently, the findings should be interpreted as preliminary. Another limitation is the absence of patient-reported outcome measures (PROMs). The inclusion of both patient- and clinician-based esthetic evaluations would have provided a more comprehensive perspective. Additionally, the assessment of interdental papillae and the potential influence of adjacent teeth on creeping attachment were not evaluated in this study. To address these limitations and obtain higher levels of evidence, future research should employ randomized, split-mouth designs with larger sample sizes. Ideally, such studies should include a control group comparing I-PRF with gold-standard root coverage techniques or with combined I-PRF and MN approaches. Moreover, given that aging can influence fibroblast activity and periodontal ligament cell properties, comparative studies across different age groups may provide valuable insights.^37-39^ Finally, implementing recent innovations—such as horizontal centrifugation, hydrophobic I-PRF tubes, and post-centrifugation blood cooling—along with histological assessments, may further strengthen the clinical relevance and biological understanding of future findings.^40,41^

## Conclusion

This study demonstrated that I-PRF application resulted in limited RC% in both phenotypes. Although the thin phenotype group showed higher RC% values in the short term compared to the thick phenotype group, this improvement was not maintained over the long term, and greater gingival stability was observed in the thick phenotype group. Furthermore, KTW increased significantly in both groups, while GT showed a significant increase, particularly in the thin phenotype group. Initial KTW positively affected recession coverage values in the short term, whereas tooth position was an important factor affecting long-term stability. Demographic factors such as age and gender did not affect clinical outcomes. Long-term follow-up studies comparing I-PRF with surgical root coverage techniques are needed. Future studies with larger sample sizes and longer follow-up periods are also required.
